# Editorial: Perioperative management and cancer outcome

**DOI:** 10.3389/fonc.2023.1251728

**Published:** 2023-08-08

**Authors:** Yiying Tao, Juan P. Cata, Weian Zeng, Jie Tian

**Affiliations:** ^1^ Department of Anesthesiology, Renji Hospital, Shanghai Jiaotong University School of Medicine, Shanghai, China; ^2^ Key Laboratory of Anesthesiology (Shanghai Jiaotong University), Ministry of Education, Shanghai, China; ^3^ Department of Anesthesiology and Perioperative Medicine, The University of Texas – MD Anderson Cancer Center, Houston, TX, United States; ^4^ Department of Anesthesiology, Sun Yat-Sen University Cancer Center, State Key Laboratory of Oncology in South China, Collaborative Innovation Center for Cancer Medicine, Guangzhou, China

**Keywords:** perioperative management, cancer outcome, prognosis, postoperative complication, prehabilitation

Perioperative management plays a critical role in determining the outcomes of patients with cancer undergoing surgery. It encompasses a comprehensive approach that involves careful planning, meticulous execution, and attentive postoperative care. The perioperative period, which includes the preoperative, intraoperative, and postoperative phases, presents numerous challenges and opportunities to optimize patient care and improve treatment outcomes. The Research Topic aimed to present some of the more recent evidence integrating clinical observations and experimental findings linking perioperative management and cancer-related outcomes.

Several studies focused on the potential long-term effects of perioperative and intraoperative pharmacological management on tumors. The influence of anesthetic approaches on cancer patients is complex. Abundant evidence from animal studies has suggested that different types of anesthetics can influence tumor progression and survival outcomes in patients with malignancies (1, 2). The impact of intraoperative low-dose dopamine administration in hepatic surgery emerges as another intriguing topic within this Research Topic. The propensity score matching analysis examining its association with survival rates in hepatocellular carcinoma patients conducted by Wang et al. highlight the potential implications of such intervention on long-term outcomes. Dexmedetomidine is a frequently used sedative during surgery. Xu et al. conducted a meta-analysis and showed the impact of dexmedetomidine in reducing systemic inflammation and postoperative cognitive dysfunction and improving recovery in patients undergoing digestive tract cancer surgery.

Importantly, the Research Topic also addresses the value of non-surgical interventions in perioperative care. Prehabilitation is a proposed modality for optimizing preoperative conditions to improve postoperative outcomes. Studies reported potential advantages for various surgical procedures (3, 4). However, according to Zhang X. et al.‘s systematic reviews and meta-analyses, rehabilitation did not significantly enhance postoperative outcomes for colorectal surgery patients when postoperative complications, length of hospital stay and functional capacity were considered.

Other studies focused on the association between specific biomarkers and long-term survival rates in patients with cancer. According to Zhang H. et al., mu-opioid receptor (MOR) expression in ovarian cancer patients undergoing surgery is not an independent predictor of worse survival but is related with high rates of perineural invasion. Furthermore, one team conducted an intensive study on perioperative management and biochemical markers of colorectal cancer (CRC) patients. The articles shed light on the significance of factors such as anemia tolerance, blood transfusions, neutrophil and white blood cell (WBC) count levels in CRC. Weng et al. demonstrated that preoperative anemia and blood transfusion increased the risk of colorectal cancer surgery recurrence, thus promoting the idea of anemia tolerability and limiting the use of blood transfusion. Considering the characteristics of CRC, which is an inflammation-related tumor characterized by the infiltration of heterogeneous immune cells into the tumor microenvironment and peripheral hematological disorders (5), they included neutrophil and WBC as variables of interest in the study. Weng et al. indicated that elevated myeloperoxidase (MPO) levels in CRC patients were substantially linked with high preoperative neutrophil counts, implying that neutrophils may be crucial participants in the mechanism connecting MPO levels with poor CRC outcomes. Also, they have found that a high preoperative WBC count was a poor prognostic indicator and was associated with an immunosuppressive microenvironment in CRC patients (Weng et al.). By dissecting the relationships between these variables and patient outcomes, researchers provide a deeper understanding of the disease’s progression and offer valuable insights for tailored perioperative management strategies.

Additionally, the articles emphasize the significance of prevention and care of postoperative complications and hospital volume-patient outcome relationships. Dai et al. identified risk factors associated with an increased incidence of postoperative pulmonary complications (PPCs) in patients aged over 60 years who underwent elective colorectal surgery. The retrospective analysis revealed that age, preoperative red blood cell distribution width, and systemic inflammatory index were independent risk factors for PPCs occurrence and emphasized the importance of early identification and management. Zhang Z. et al. reviewed the literature and noted that the novel tumor suppressor esophageal cancer-related gene-4 (ECRG4) could be used in the treatment of both tumors and arrhythmias, identifying a new possible strategy to reduce the perioperative cardiovascular adverse events in patients with esophageal cancer and gastric cancer. Lei et al. suggested that centralized management of esophageal cancer surgery, while beneficial for patient survival, should ideally not exceed the identified hospital volume threshold, providing evidence-based insights for improved patient care and optimized resource allocation. These findings open doors for further exploration and potential interventions that positively influence patient prognosis.

The collective findings presented within this Research Topic, “*Perioperative management and cancer outcome*,” contribute significantly to our understanding of the intricate relationship between perioperative care and cancer prognosis. The insights derived from these articles underscore the importance of personalized, multidisciplinary approaches that consider specific biomarkers, tailored interventions, and risk factor identification. Healthcare professionals can refine perioperative management strategies and optimize cancer treatment outcomes by recognizing the potential impact of factors such as anemia tolerance. Furthermore, the significance of non-surgical interventions, including prehabilitation, targeted pharmacological approaches, and suitable hospital volume, highlights the potential for holistic patient care.

Advancements in perioperative management have the power to significantly influence long-term cancer outcomes. As editors, researchers, and healthcare professionals, let us harness the knowledge presented within this Research Topic to enhance our understanding and implementation of effective perioperative care protocols.

## Author contributions

YT, JPC, WZ and JT: concept and design. YT: drafting of the manuscript. JPC, WZ and JT: critical revision of the manuscript for important intellectual content, administrative, and supervision. All authors contributed to the article and approved the submitted version.
